# Comparable incidence of periprosthetic tibial fractures in cementless and cemented unicompartmental knee arthroplasty: a systematic review and meta-analysis

**DOI:** 10.1007/s00167-021-06449-3

**Published:** 2021-02-02

**Authors:** Joost A. Burger, Tjeerd Jager, Matthew S. Dooley, Hendrik A. Zuiderbaan, Gino M. M. J. Kerkhoffs, Andrew D. Pearle

**Affiliations:** 1 Department of Orthopaedic Surgery and Computer Assisted Surgery Center, Hospital for Special Surgery, Weill Medical College of Cornell University, 535 East 70th St, New York, NY 10021 USA; 2 Department of Orthopaedic Surgery, Spaarne Gasthuis, Hoofddorp, The Netherlands; 3 Department of Orthopaedic Surgery, Noordwest Ziekenhuisgroep, Alkmaar, The Netherlands; 4 Department of Orthopedic Surgery, Amsterdam Movement Sciences (AMS), Amsterdam University Medical Centers, Amsterdam, The Netherlands

**Keywords:** Periprosthetic fractures, Tibial plateau fractures, Complications, Failure modes, Unicompartmental knee arthroplasty, Partial knee replacement, UKA, PKR

## Abstract

**Purpose:**

*(I)* To determine the incidence of periprosthetic tibial fractures in cemented and cementless unicompartmental knee arthroplasty (UKA) and (II) to summarize the existing evidence on characteristics and risk factors of periprosthetic fractures in UKA.

**Methods:**

Pubmed, Cochrane and Embase databases were comprehensively searched. Any clinical, laboratory or case report study describing information on proportion, characteristics or risk factors of periprosthetic tibial fractures in UKA was included. Proportion meta-analysis was performed to estimate the incidence of fractures only using data from clinical studies. Information on characteristics and risk factors was evaluated and summarized.

**Results:**

A total of 81 studies were considered to be eligible for inclusion. Based on 41 clinical studies, incidences of fractures were 1.24% (95%CI 0.64–2.41) for cementless and 1.58% (95%CI 1.06–2.36) for cemented UKAs (9451 UKAs). The majority of fractures in the current literature occurred during surgery or presented within 3 months postoperatively (91 of 127; 72%) and were non-traumatic (95 of 113; 84%). Six different fracture types were observed in 21 available radiographs. Laboratory studies revealed that an excessive interference fit (press fit), excessive tibial bone resection, a sagittal cut too deep posteriorly and low bone mineral density (BMD) reduce the force required for a periprosthetic tibial fracture to occur. Clinical studies showed that periprosthetic tibial fractures were associated with increased body mass index and postoperative alignment angles, advanced age, decreased BMD, female gender, and a very overhanging medial tibial condyle.

**Conclusion:**

Comparable low incidences of periprosthetic tibial fractures in cementless and cemented UKA can be achieved. However, surgeons should be aware that an excessive interference fit in cementless UKAs in combination with an impaction technique may introduce an additional risk, and could therefore be less forgiving to surgical errors and patients who are at higher risk of periprosthetic tibial fractures.

**Level of evidence:**

V.

## Introduction

Unicompartmental knee arthroplasty (UKA) is a well-established treatment for patients with isolated compartmental knee arthritis. Advantages of UKA over total knee arthroplasty (TKA) include reduced morbidity and mortality, preservation of normal knee kinematics and faster recovery [35, 49, 59]. However, national registry data have shown lower revision rates after TKA in comparison to UKA [49, 66]. Reasons for UKA revision include aseptic loosening, malalignment, progression of osteoarthritis, instability, infection and periprosthetic fractures [49, 66].

Periprosthetic fractures represent a complex complication with serious consequences in UKA and have been associated with increased mortality and morbidity [26]. The periprosthetic fractures in UKA are most commonly reported on the tibial side (approximately 87%) [66]. Although these periprosthetic tibial fractures are relatively rare compared to other complications in UKA, recent registry-based studies have shown an increased rate of periprosthetic fractures in cementless UKAs compared to cemented UKAs [49, 63]. Since the interest of cementless fixation for UKAs is expected to increase, the rate of periprosthetic fractures may increase as well [49, 63]. However, registry-based studies may not provide reliable information about all fractures, as some periprosthetic fractures are internally fixed and the components are not revised or are treated conservatively. Another common limitation of registry-based studies is that tibial and femoral periprosthetic fractures are not reported separately. This stresses the need for a thorough evaluation of the incidence of periprosthetic tibial fractures in cemented and cementless UKAs using clinical studies. Furthermore, there is a lack of studies providing an overview of the available evidence on characteristics and risk factors of periprosthetic tibial fractures in UKA to gain a better understanding and awareness.

Therefore, the primary study aim was to estimate the incidence of periprosthetic tibial fractures in cemented and cementless UKA using clinical studies. Secondarily, relevant studies were systematically reviewed to summarize characteristics and risk factors of periprosthetic tibial fractures in UKA. Based on earlier large case series of both cemented and cementless UKAs reporting no non-traumatic periprosthetic tibial fractures [62, 68], it was hypothesized that comparable low incidences of periprosthetic tibial fractures can be achieved as long as surgeons are aware of factors that could increase the risk.

## Methods

### Search strategy

This systematic review with meta-analysis was conducted according to the PRISMA guidelines [65]. Medline, Cochrane and Embase databases were comprehensively searched on 28 May 2020. The database search included several combinations of key terms: “unicompartmental”, “knee”, “arthroplasty”, “failure”, “complication”, “survival”, “survivorship”, “revision”, “reoperation”, “fracture” and “collapse”. The search was, however, limited to English language studies published since 2000.

After duplicates were excluded, titles and abstracts were screened by two independent reviewers (*** & ***). Subsequently, full texts of the potential studies were carefully assessed by the two reviewers to confirm study eligibility. To be eligible, the study needed to contain information on proportion, characteristics and/or risk factors of periprosthetic tibial fractures in UKA. Clinical studies with information on fixation type and proportion were used to estimate incidences. For information regarding characteristics and/or risk factors, any study design was considered eligible, including case reports and laboratory studies. Although case reports and laboratory studies constitute low-level evidence, a systematic review of such studies can provide a better understanding and awareness of tibial plateau fractures in UKA. Studies were excluded if they reported on bicompartmental UKAs, used the same database, were reviews, registry-based studies, commentaries or abstracts. References of the included studies were checked for any missing studies. Any disagreements on study eligibility were resolved through consultation of the third reviewer (***).

### Data collection and analysis

Data extraction was entered in predefined spreadsheets by two independent reviewers. First author, publication year and study design were reported for each study. Total number of UKAs, number of fractures and fixation type were collected only from clinical studies for the analysis of incidence. To identify potential risk factors, characteristics of patients with and without periprosthetic tibial fractures were collected from clinical studies and compared. For example, body mass index (BMI) of patients with and without fractures were compared. Both clinical studies and case reports were used to evaluate characteristics of periprosthetic tibial fractures (time of fracture in relation to UKA, fracture mechanism [traumatic or non-traumatic], fracture type, type of treatment). Time of fracture in relation to UKA was classified into the following time-points: during surgery, within 3 months postoperatively, between 4 and 12 months postoperatively and after 1 year postoperatively. Schematic drawings were used to present the fracture types found on available radiographs. Causes of fractures considered by authors from each study were evaluated and summarized. Finally, conclusions of laboratory studies were presented.

### Methodological quality assessment

Different tools for methodological quality assessment were used depending on study design.

The National Institutes of Health (NIH) checklist was used for all clinical studies [67],The Case Report (CARE) checklist was used for case reports [29],and the Quality Appraisal for Cadaveric Studies (QUACS) checklist [90] was used for cadaveric studies. A score was provided for each article (poor, fair or good). The assessment was performed by two independent reviewers (*** & ***) and disagreements of the level of study quality were resolved through consultation of the third reviewer (***).

### Statistical analyses

Incidence of periprosthetic tibial fractures was calculated as the number of fractures divided by the total number of UKAs from each clinical study. These data were combined via proportion meta-analysis [94]. This is a tool to calculate an overall proportion from studies reporting a single proportion. Combined proportions were determined for cementless and cemented UKAs. A subgroup analysis was performed for cementless and cemented Oxford Partial Knee Implants. Effect sizes and 95% Confidence Intervals (CI) were determined using a random-effects model by the back-transformation of the weighted mean of the logit-transformed proportions with Dersimonian weights. Characteristics between patients with and without periprosthetic tibial fractures were compared using the chi-square test for categorical variables and independent t test for continuous variables. All analyses were performed with R version 4.0.0 (R Foundation for Statistical Computing, Vienna, Austria).

## Results

**Fig. 1:**
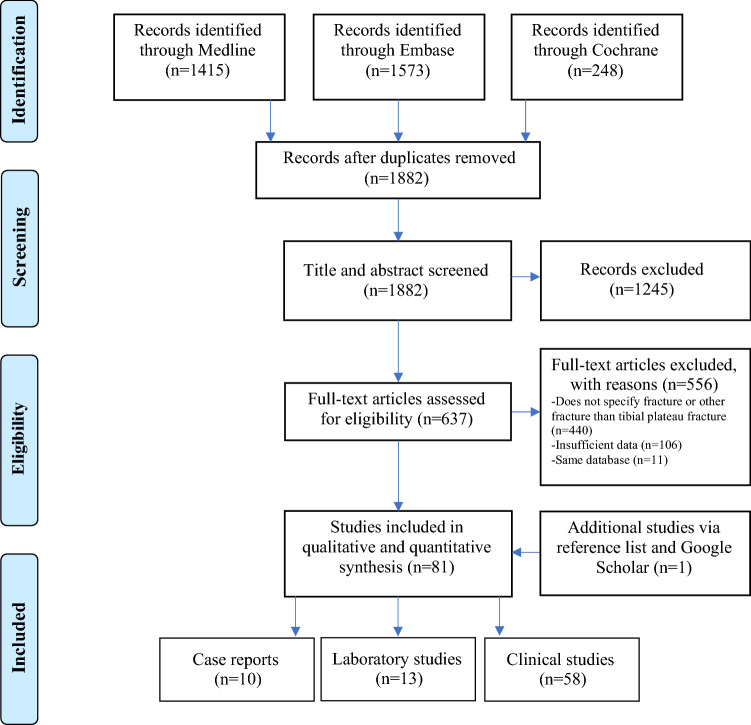
Preferred Reporting Items for Systematic Reviews and Meta-Analyses flow diagram

A total of 81 studies were included (Fig. 1). Fifty-eight (72%) were clinical studies consisting of 30 retrospective case series (52%) [1–6, 8–11, 14, 27, 31, 36, 37, 43–45, 47, 48, 53, 54, 70, 73, 83, 85, 88, 91, 93, 96], 14 prospective case series (26%) [7, 17, 18, 32, 51, 55–58, 61, 77, 78, 86, 95], seven retrospective cohort studies (12%) [13, 24, 25, 46, 50, 59, 72], four prospective cohort studies (7%) [28, 30, 84, 89] and three randomized controlled trials (5%) [22, 23, 33]. Ten (12%) studies were case reports [15, 40, 52, 60, 69, 74, 81, 82, 87, 92]. Thirteen (16%) were laboratory studies, of which four (31%) used sawbones [16, 20, 39, 64], four (31%) finite element models [41, 42, 75, 76], three (23%) human cadavers [21, 79, 80] and two (15%) a combination of finite element models with sawbones [19, 71]. The quality of studies was considered to be good in 54 (67%) studies, fair in 26 (32%) studies, and poor in one (1%) study. Table 1 summarizes the conclusions and quality assessment of the laboratory studies. Appendix 1 and 2 summarize the data extraction and quality assessment of the case reports and clinical studies, respectively.

**Table 1:** Summary of laboratory studies

Study	Country	Study type	Implant	Summary	Study quality *
Campi et al. [16]	UK	Sawbone	Oxford (Biomet)	This study suggests that decreasing the press fit of the tibial keel of the cementless UKA would significantly decrease the push-in force required to insert the tibial component (and so decrease the risk of fracture), without reducing the pull-out force and therefore ensuring the same level of primary stability	Good
Chang et al. [19]	Taiwan	FE model & Sawbone	Miller-Galante II, cemented (Zimmer)	This study suggests that in UKA, rounding the resection corner during preparation of the tibial plateau decreases the strain on tibial bone and avoid degenerative remodeling, in comparison to a standard rectangular corner. This modified surgical technique using a predrilled tunnel through the tibia prior to cutting could avoid extended vertical saw cutting errors	Good
Clarius et al. [20]	Germany	Sawbone	Oxford (Biomet)	This study suggests several sawing errors can occur during preparation of the tibial plateau (extended vertical cuts which may reduce the stability of the medial tibial plateau, extended horizontal cuts, perforation of the posterior cortex) and femoral condyle (ascending cut at the posterior femoral condyle) in UKA, especially with inexperienced surgeons	Good
Clarius et al. [21]	Germany	Cadaver	Oxford UKA (Biomet)	This study suggests that extended sagittal saw cuts in UKA weaken the tibial bone structure and increase the risk of periprosthetic tibial plateau fractures. In addition, this study showed that UKA patients with low BMD are at higher risk, as the fracture load is dependent on the bone density	Good
Iesaka et al. [41]	Japan	FE model	NR	In UKA, placing the tibial component in slight valgus inclination is preferred to varus or square inclination as it results in more even stress distributions	Fair
Inoue et al. [42]	Japan	FE model	Metal-backed tibia, cemented	This study suggests that the risk of medial tibial condylar fractures in UKA increases with increasing valgus inclination of the tibial component and with increased extension of the sagittal cut in the posterior tibial cortex	Good
Mohammad et al. 2018	UK	Sawbone	Oxford, cementless (Zimmer Biomet)	This study suggests to use a new wider and deeper keel cut saw blade in UKA as it decreases the risk of tibial fracture compared to the standard keel cut saw blade, with no compromise in fixation	Good
Sasatani et al. 2019	Japan	FE model	Persona (Zimmer Biomet)	This study suggests that the optimal alignment of the tibial implant in UKA is the middle position the coronal plane and the original posterior inclination in the sagittal plane	Good
Sawatari et al. 2005	Japan	FE model	SCR UKA, metal-backed tibia, cemented (Stryker)	This study suggests that in UKA, placing the tibial component in slight valgus inclination is recommended due to reduced stress on tibial cancellous bone, in comparison with varus or square inclination. However, excessive posterior slope should be avoided	Good
Seeger et al. [79]	Germany	Cadaver	Oxford cemented & cementless (Biomet)	The risk for periprosthetic tibial plateau fractures is higher with cementless UKA than cemented UKA, especially in patients with poor bone quality	Good
Seeger et al. [80]	Germany	Cadaver	Oxford (Biomet)	Concerning the treatment of periprosthetic tibial plateau fractures in UKA, angle-stable plates provides better initial stability than fixation with cannulated screws	Good
Pegg et al. [71]	UK	FE model and Sawbone	Oxford (Biomet)	This study suggests that excessive resection depth and making the vertical cut too deep posteriorly increase the risk for periprosthetic tibial fractures in UKA	Good
Houskamp et al. [39]	USA	Sawbone	Metal-backed fixed-bearing (Stryker)	In UKA, tibial resections beyond 5.82 mm increase the risk of periprosthetic fractures	Good

*UKA* unicompartmental knee arthroplasty; *NR* not reported

*Quality Appraisal for Cadaveric Studies (QUACS) Scale was used as a quality assessment tool

### Incidence of fixation type

**Fig. 2:**
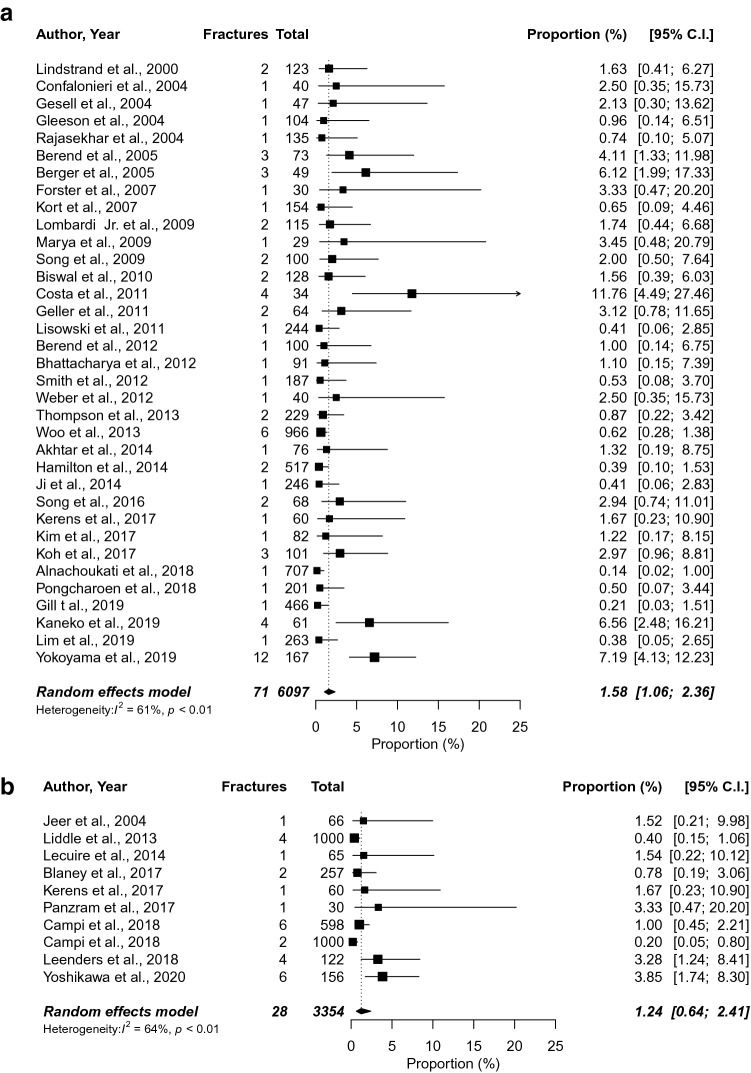
Proportion meta-analysis to estimate the incidence of fractures in cemented (**a**) and cementless (**b**) unicompartmental knee arthroplasty

**Fig. 3:**
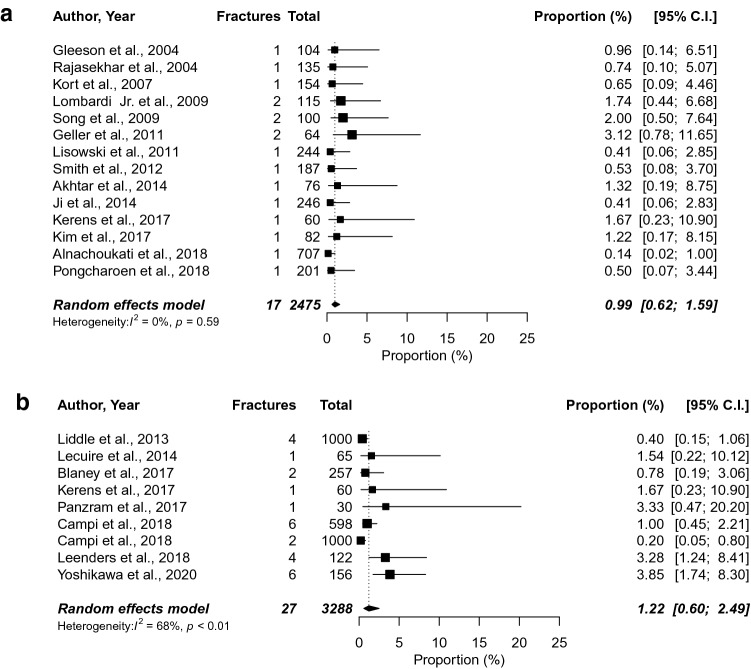
Proportion meta-analysis to estimate the incidence of fractures in cemented (**a**) and cementless (**b**) Oxford Partial Knee unicompartmental knee arthroplasty

The incidence of each fixation type was determined using 44 clinical studies [1, 3, 5–10, 17, 18, 22, 23, 28, 30–33, 36, 43–46, 48, 50, 51, 53–59, 61, 70, 72, 73, 83–86, 89, 91, 93, 96], leading to a incidence of 1.24% (95% CI 0.64–2.41) for cementless and 1.58% (95% CI 1.06–2.36) for cemented UKAs (Fig. 2). Subgroup analysis for the Oxford Partial Knee implants was performed using 21 clinical studies [1, 3, 10, 17, 18, 30, 33, 44, 46, 48, 51, 53–55, 58, 59, 70, 72, 73, 83, 85, 96], resulting in an incidence of 1.22% (95% CI 0.60–2.49) for cementless and 0.99% (95% CI 0.62–1.59) for cemented fixation (Fig. 3).

### Characteristics

A total of 202 periprosthetic tibial fractures in UKA were reported in 58 clinical studies [1–4, 6–11, 13, 14, 17, 18, 22–25, 27, 28, 30–33, 36, 37, 43–48, 50, 51, 53–59, 61, 70, 72, 73, 77, 78, 83–86, 88, 89, 91, 93, 95, 96] and ten case reports [15, 40, 52, 60, 69, 74, 81, 82, 87, 92]. The time of fracture was noted for 127 fractures. Twenty-three fractures (18%) occurred during the operation, 68 (54%) presented within 3 months postoperatively, 19 (15%) presented between 4 and 12 months postoperatively, and 17 (13%) presented after 1 year postoperatively. Fracture mechanism was reported for 113 fractures with 95 (84%) being non-traumatic.

**Fig. 4:**
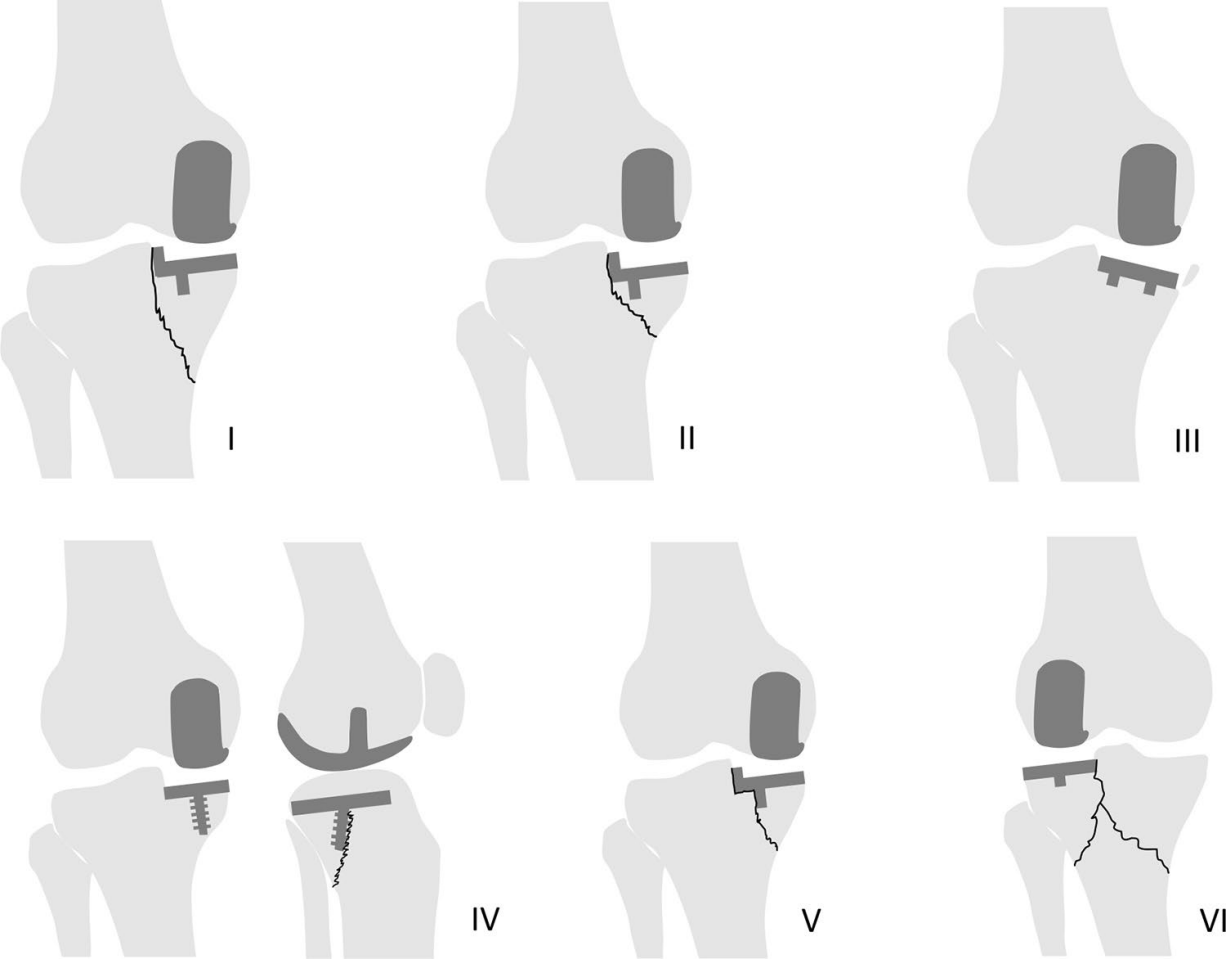
Periprosthetic tibial fracture types in unicompartmetnal knee arthroplasty (UKA) seen on radiographs. **I–II**: Fracture line extending from the corner of the tibial resection to the medial cortex, resulting in a large (**I**) or small (**II**) medial plateau fracture. These fracture lines were identified on the anteroposterior (AP) view in patients with different implant designs. **III**: Varus subsidence or anterior subsidence of the tibia component, resulting in a small medial fragment fracture. These fractures were identified on the AP view. **IV**: Fracture line extending from the screw fixation to the posterior cortex, resulting in a posteromedial plateau fracture. The fracture line could not be identified on the AP view but only on the lateral view in a patient with a cementless fixed-bearing UKA with screw fixation. **V**: Fracture line extending from the tibial keel to the medial cortex, resulting in a medial plateau fracture. These fracture lines were identified on the AP view in patients with Oxford Partial Knee implants. **VI**: Two fracture lines extending from the corner of the tibial resection to the medial and lateral cortex after traumatic event six years postoperatively, resulting in a bicondylar plateau fracture. The fracture line was identified on the AP view in a patient with a lateral UKA

Twenty-one fractures (10%) had good-quality radiographs to assess the location of the fracture line [6, 14, 33, 40, 45, 48, 52, 69, 74, 81, 85, 87, 88, 92]. Schematic drawings of the different fracture types are displayed in Fig. 4.

Based on information from 167 fractures, 85 (51%) periprosthetic tibial fractures were treated with TKA (with metal augmentation and/or tibial stem extension), 38 (23%) with ORIF, and 44 (26%) with conservative treatment. Authors reported that eight fractures, initially treated conservatively, underwent a subsequent TKA; six fractures, initially treated with ORIF, underwent a subsequent TKA; one fracture, initially treated conservatively, underwent ORIF; and one fracture, initially treated conservatively, underwent ORIF and eventually needed a TKA.

### Risk factors

Factors related to periprosthetic tibial fractures in UKA were analyzed using 23 clinical studies Table 2 [1, 8–10, 13, 18, 23–25, 28, 31, 32, 37, 43, 47, 48, 57, 61, 86, 89, 91, 93, 96]. Fractures were associated with increased BMI (*p* = 0.017), advanced age (*p* = 0.003), decreased bone mineral density (BMD) (*p* = 0.030), female gender (*p* = 0.011), increased postoperative tibia-femoral alignment (*p* = 0.0120) and a very overhanging medial tibial condyle (< 0.001). The definition of a very overhanging medial tibial condyle was based on the medial eminence line (MEL) described by Yoshikawa et al. [96]. The MEL is a line drawn on preoperative radiographs, that is parallel to the tibial axis passing through the tip of medial intercondylar eminence. If this line passes medial to the medial cortex of the tibia, knees were classified as having a very overhanging medial tibial condyle. Fractures were not associated with the postoperative level of patient activity (*p* = 0.976) or with the tibial component alignment angle in the coronal plane (*p* = 0.130).

**Table 2:** Results of the comparison between UKAs without and with fractures

	No. of clinical studies	Group	No. of knees	Mean ± SD or %	*P* value^§^
Body mass index (kg/m^2^)	4	UKAs without fractures	1379	26.3 ± 6.8*	0.017
		UKAs with fractures	12	31.0 ± 6.8	
Age (yrs)	14	UKAs without fractures	2701	64.4 ± 9.2*	0.003
		UKAs with fractures	24	70.0 ± 9.2	
Bone mineral density (g/m^2^)	1	UKAs without fractures	155	0.73 ± 0.10	0.030
		UKAs with fractures	12	0.65 ± 0.16	
Tibial component angle (°)	1	UKAs without fractures	155	4.19 ± 2.94	0.130
		UKAs with fractures	12	2.83 ± 2.69	
Postoperative Tibia-femoral Angle (°)	1	UKAs without fractures	155	176.5 ± 3.6	0.012
		UKAs with fractures	12	179.3 ± 3.3	
Gender (Female/Male)	20	UKAs without fractures	5910	67%/33%	0.011
		UKAs with fractures	58	83%/17%	
Activity level (High/Low) ^#^	1	UKAs without fractures	566	20%/80%	0.976
		UKAs with fractures	10	20%/80%	
Very overhanging medial tibial condyle (Yes/No) ^†^	1	UKAs without fractures	150	12%/88%	< 0.001
		UKAs with fractures	6	67%/33%	

^§^Chi square test was used for categorical variables and the independent *t* test for continuous variables

^#^Patients with an UCLA (University of California Los Angeles) activity score > 6 were classified as high

*The weighted mean of the overall UKA population with the same standard deviation as the tibial plateau fracture cases was used to allow for a fair comparison. This means this is an estimation and not the exact mean with standard deviation of the UKAs without fractures

^†^Very overhanging medial tibial condyle was defined as a medial eminence line outside the medial cortex of the tibial shaft as described by Yoshikawa et al.[95]

### Authors considerations

Authors reported their considerations of cause of fracture in 36 clinical studies [1, 2, 4–11, 13, 14, 17, 18, 23, 30, 31, 33, 36, 37, 43–45, 54, 55, 57, 61, 70, 84, 85, 88, 89, 91, 93, 95, 96] and nine case reports [15, 40, 52, 60, 69, 74, 81, 82, 87, 92] (Table 3).

**Table 3:** Factors associated with periprosthetic tibial fractures considered by authors

Implant and surgical factors
Excessive postoperative alignment angle
Pin placement (excessive pins, not predrilled, too close to medial tibial cortex)
Excessive tibial bone resection
Vertical saw cut too distal in posterior tibial cortex
Excessive posterior slope
Error in keel preparation
Learning curve/introduction of new implant
Limited instrumentation
Not enough medialization of the tibial component to tibial spine
Tibial peg hole drilled too deeply
All-polyethylene design
Tibial subsidence or collapse
Undersizing or oversizing of tibia component
Forceful impaction
**Patient factors**
Infection
Osteoporosis
Overweight
Small tibial size
Very overhanging medial tibial condyles
Trauma
**Rehabilitation factor**
Weightbearing too early

## Discussion

The main study finding was that the incidence of periprosthetic tibial fractures in cemented and cementless UKA was comparable. However, experimental evidence showed that excessive interference fit (press fit), excessive resection depth, making the sagittal cut too deep posteriorly, and low BMD reduces the load required for a periprosthetic tibial fracture to occur. Furthermore, clinical studies revealed that patients with fractures were more often female, of older age, exhibited higher BMI and postoperative alignment angles, had lower BMD and had very overhanging medial tibial condyles.

Contrarily to the main finding of this study, two recent registry-based studies showed higher rates of periprosthetic fractures in cementless compared to cemented Oxford Partial Knee implants [49, 63], raising some concerns regarding a keel design in cementless techniques. Campi et al. demonstrated that fixation of the cementless mobile-bearing Oxford UKA is ensured by the interference fit [18]. However, an excessive interference increases the assembly load required to push-in the component potentially introducing a splitting force during impaction (type V fracture) [16]. As this interference fit, combined with an impaction technique, could introduce an additional risk factor for fractures, the cementless Oxford Partial Knee implant may be less forgiving to surgical errors and patients who are at higher risk of periprosthetic tibial fractures.

Several surgical errors have been proposed by authors to cause periprosthetic tibial fractures in UKA Table (3). Only a few authors have supported their conclusion with experimental evidence. Laboratory studies showed a vertical saw cut too distal in the posterior tibial cortex and excessive tibial bone resection reduces the load required for a fracture to occur [20, 21, 39, 71]. Additionally, laboratory studies on the role of tibial component alignment suggested valgus alignment and an excessive posterior slope should be avoided [41, 42, 76]. Other authors based their conclusions on radiographic or intraoperative findings. Radiographs revealed that fracture lines went through multiple pinholes of the extramedullary tibial guide (type II fracture) [15]. One author reported that a fracture occurred due to breaching the posterior cortex while using a tibial gouge for keel preparation in Oxford Partial Knee implants (type V fracture) [82]. Furthermore, one fracture occurred after breaching the tibial cortex with the screw to fixate a cementless fixed-bearing UKA (type VI) [87]. These findings indicate that surgical actions that weaken cortical bone or reduce the bony area under the tibial component increase the risk of fracture. However, more studies evaluating fractures under different conditions in UKA are necessary to understand the main pathologic elements of periprosthetic tibial fractures.

It was further noted that female gender, higher BMI and age, osteoporosis, excessive postoperative alignment angles and a very overhanging medial tibial condyle could contribute to the occurrence of periprosthetic tibial fractures in UKA. The relationship with greater age and osteoporosis is not surprising as fractures have been directly linked to these factors [12]. The higher proportion of periprosthetic tibial fractures in females compared to males may be due to higher rate of osteoporosis[12], the smaller average size of tibial plateaus [97] and the higher likelihood of having very overhanging medial tibial condyles [38, 96] in females. The two latter reasons reduce the bone volume to support the tibial component which may increase the risk of fracture. As such, surgeons should avoid large tibial resections as well as peripheral positioning [39], especially in those with already little bone volume to support the tibial component. Further, the relationship of higher BMI and excessive postoperative alignment angles with periprosthetic tibial fractures may be explained by the excessive loads placed on the small tibial surface [40, 74, 84]. In addition, small medial femoral condyles needing small components might also be a risk factor leading to overload because of smaller contact areas at the medial tibial surface [34].

Despite surgeons should be aware of potential risk factors, current evidence underlines developments in instrumentation and implants can minimize fracture risk. Chang et al. showed a modified technique using a predrilled tunnel through the tibia prior to cutting could avoid extended vertical saw cut errors [19]. Campi et al. suggested the optimal interference fit for good implant stability and minimal risk of fracture is between 0.5 mm and 0.7 mm [16]. Mohammad et al. reported improvements in instrumentation that widen the keel slot could reduce the risk of tibial fractures in cementless Oxford Partial Knee implants without compromising fixation [64]. Some authors suggested to change the depth of the tibial keel in very small cementless Oxford Partial Knee components as the depth of the keel is currently the same in all components, increasing the risk of fracture [38]. Vardi et al. reported that a change was made to the shape and size of the tibial keel of the Alphanorm implant due to high rates of periprosthetic tibial fractures [88].

This study revealed that most of periprosthetic tibial fractures occurred intraoperatively or within 3 months of surgery and were non-traumatic. Studies of intraoperative fractures described that operative damage in combination with the impaction of the tibial component caused the tibial bone to fracture. The postoperative fractures within 3 months may be associated with operative damage and repetitive stress on the bone during daily activities such as walking and stair climbing. Fractures that presented after 3 months were mostly associated with traumatic events, excessive weight, osteoporosis, infection, all-polyethylene designs and tibial component malposition.

Furthermore, a classification of periprosthetic tibial fracture types was presented. As only 10% of all fractures could be used in the classification, the incidence and completeness of fracture types in UKA remain unknown. However, presented paths of fractures could explain the high-risk fracture regions. For example, the type I fracture not only suggest that an extended sagittal cut posteriorly can initiate a fracture, but indicate that risk of fracture propagation can be increased by placing pins from the extramedullary tibial guide within fracture line regions.

Some limitations of this study should be noted. First, the pooled estimated incidences of fractures were not adjusted for the follow-up period. However, almost all clinical studies had a minimum follow-up of one year and thus included the period when the majority of fractures occurred. Second, poor reporting on characteristics of fractures may have biased the results. Third, not all risk factors for fractures in UKA mentioned by authors have been verified with clinical data, and therefore might be subjective. Also, it cannot be clarified which risk factors verified with clinical data were independently related to periprosthetic tibial fractures as the findings were based on unadjusted analyses. Fourth, to analyze whether increased BMI and age were related to fracture cases, the weighted mean of the overall UKA population was used with the same standard deviation as those of the periprosthetic tibial fracture cases. Although this approach can be considered a fair approximation, the statistical difference for BMI and age between UKAs with and without fractures may have been underestimated. Finally, this study did not focus on the diagnostics and treatment of periprosthetic tibial fracture in UKA. However, based on the current search, three studies have currently evaluated the management of periprosthetic tibial fractures in UKA [14, 80, 91]. Treatments of the included fracture cases were reported to give a complete overview. Despite the aforementioned limitations, this is the first study evaluating the incidence of periprosthetic tibial fractures in cemented and cementless UKAs and providing an overview of the available evidence on periprosthetic tibial fracture in UKA.

## Conclusion

The incidence of periprosthetic tibial fractures in cementless UKAs can be similar to those seen in cemented UKAs. However, surgeons should be aware that an excessive interference fit for cementless UKAs in combination with an impaction technique may introduce an additional risk, and may, therefore, be less forgiving to surgical errors and patients who are at higher risk of periprosthetic tibial fractures. While findings of this study raise awareness about periprosthetic tibial fractures in UKA, this study also highlights the importance of improvements in instrumentation and implants to prevent periprosthetic tibial fractures in future practices.

## Appendix

See Tables

**Table 4:** Summary of case reports

Study	Country	No. cases	Time point	UKA Laterality	Trauma	Gender	BMI (kg/m^2^)	Osteop	Age (year)	Implant	Cement	Treatment	Study Quality*
Brumby et al. [15]	Australia / USA	4	6 wks 3 mo 3 mo 3 wks	Medial Medial Medial Medial	No No No No	Female Female Male Female	NR NR NR NR	Yes NR NR NR	72 57 65 62	NR, Fixed bearing NR NR NR	Yes Yes Yes Yes	Conservative > TKA Conservative > TKA Conservative > TKA TKA	Fair
Rudol et al. [74]	UK	1	2 wks	Medial	No	Male	NR	NR	80	Oxford Phase 3 (Biomet)	Yes	ORIF	Fair
Lu et al. 2019	China	2	3 wks 2 wks	Medial Medial	No No	Male Female	NR NR	Yes Yes	70 72	NR NR	NR NR	ORIF Conservative	Fair
Seon et al. [81]	South Korea	2	3 wks 5 wks	Medial Medial	No No	Female Female	29.6 32.1	Yes Yes	65 68	Miller Galante (Zimmer) Miller Galante (Zimmer)	Yes Yes	ORIF TKA	Fair
Sloper et al. [82]	UK	1	Intraop	Medial	No	Male	NR	No	58	Oxford (Biomet)	-Yes	-ORIF	Fair
Kumar et al. [52]	Canada	1	6 years	Lateral	Yes	Female	NR	Yes	70	NR	Yes	TKA	Good
Van Loon et al. [87]	Belgium	3	6 days Intraop Intraop	Medial Medial Medial	No No No	Female Female Female	NR NR NR	NR NR NR	62 57 45	Accuris (Smith & Nephew) NR Profix (Smith & Nephew)	NR NR NR	ORIF > TKA Conservative > TKA TKA	Fair
Yang et al. [92]	Singapore	2	5 mo 3 mo	Medial Medial	No No	Female Male	NR NR	NR NR	60 71	PFC Sigma (Johnson Johnson) PFC Sigma (Johnson Johnson)	NR NR	TKA Conservative	Fair
Pandit et al. [69]	UK	8	Intraop 6 wks 10 wks Intraop 4 wks 12 wks 24 wks 16 wks	Medial Medial Medial Medial Medial Medial Medial Medial	No No No No No No No No	Female Male Female Female Male Male Female Female	NR NR NR NR NR NR NR NR	NR NR NR NR NR NR NR NR	72 65 55 73 82 67 76 67	Oxford (Biomet) Oxford (Biomet) Oxford (Biomet) Oxford (Biomet) Oxford (Biomet) Oxford (Biomet) Oxford (Biomet) Oxford (Biomet)	NR NR NR NR NR NR NR NR	Conservative Conservative ORIF Conservative > ORIF > TKA Conservative > TKA Conservative > TKA TKA TKA	Fair
Yuk Wah et al. 2018	China	1	4 wks	Medial	No	F emale	NR	yes	75	ZUK (Zimmer Biomet)	Yes	ORIF > TKA	Good

*UKA* unicompartmental knee arthroplasty; *BMI* body mass index; *ORIF* open reduction internal fixation; *NR* not reported; *TKA* total knee arthroplasty

*Quality Appraisal for Cadaveric Studies (QUACS) checklist was used as a quality assessment tool

4,

**Table 5:** Summary of clinical studies

Study	Country	UKA population				Fracture cases											Study design	Quality*
		Baseline (Knees)	Mean BMI	Mean Age	Female (%)	No. cases	Time point	UKA Laterality	Trauma	Gender	BMI (kg/m^2^)	Osteop	Age (yrs)	Implant	Cement	Treatment		
Akhtar et al. [1]	UK	76	30	64	58	1	-2 mo	-Medial	-Yes	-Male	-29.8	-NR	-78	-Oxford (Biomet)	-Yes	-ORIF	Case series, retrospective	Good
Aleto et al. [2]	USA	NR	NR	NR	NR	15	-16 mo -14 NR	-15 Medial	-15 NR	-15 NR	-15 NR	- 15 NR	- 15 NR	-15 NR	-15 NR	-15 TKA	Case series, retrospective	Fair
Alnachoukati et al. [3]	USA	707	32	64	45	1	-9.6 yrs	-Medial	-NR	-NR	-NR	-NR	-NR	-Oxford Phase 3 (Biomet)	-Yes	-NR	Case series, retrospective	Good
Argenson et al. [4]	France	38	26	61	62	1	-11 mo	-Lateral	-No	-NR	-NR	-NR	-NR	-Fixed-bearing, metal-backed	-Yes	-TKA	Case series, retrospective	Good
Berend et al. [5]	USA	100	30	68	70	1	-2 yrs	-Lateral	-Yes	-NR	-NR	-NR	-NR	-Repicci II/VanguardM (Biomet)	-Yes	-ORIF	Case series, retrospective	Good
Berend et al. [6]	USA	73	32	66	77	3	-1 mo -NR -NR	-Medial -Medial -Medial	-No -No -No	-Female -NR -NR	-24.9 -NR -NR	-NR -NR -NR	-72 -NR -NR	-Fixed-bearing -Fixed-bearing -Fixed-bearing	-Yes -Yes -Yes	-NR -NR -NR	Case series, retrospective	Good
Berger et al. [7]	USA	49	NR	68	67	3	-Intraop -Intraop -Intraop	-Medial -Medial -NR	-No -No -No	-NR -NR -NR	-NR -NR -NR	-NR -NR -NR	-NR -NR -NR	-Miller-Galante (Zimmer) -Miller-Galante (Zimmer) -Miller-Galante (Zimmer)	-Yes -Yes -Yes	-Conservative -ORIF -Conservative	Case series, prospective	Good
Bhattacharya et al. [8]	UK	91	NR	68	58	1	-31 mo	-Medial	-Yes	Male	-NR	-NR	-65	-Preservation (DePuy)	-Yes	TKA	Case series, retrospective	Good
Biswal et al. [9]	Australia	128	29	68	49	2	-10 mo -50 mo	-Medial -Medial	-No -Yes	-NR -NR	-36.0 -40.1	-No -Yes	-58 -57	-Allegretto (Zimmer) -Allegretto (Zimmer)	-Yes -Yes	-TKA -TKA	Case series, retrospective	Good
Blaney et al. [10]	UK	257	30	65	48	2	-2 wks -13 mo	-Medial -Medial	-No -Yes	-Female -Male	-NR -NR	-NR -NR	-NR -NR	-Oxfort (Biomet) -Oxfort (Biomet)	-No -No	-ORIF -ORIF	Case series, retrospective	Good
Bohm et al. [11]	Austria	278	NR	NR	NR	1	-1 wk	-Medial	-No	-NR	-NR	-NR	-NR	-NR	-NR	-TKA	Case series, retrospective	Good
Bonutti et al. [13]	USA	80	33	66	45	1	-9 mo	-Medial	-No	-Male	-NR	-NR	-NR	-Fixed-bearing	-NR	-TKA	Cohort study, retrospective	Good
Brown et al. [14]	USA	2464	NR	NR	NR	16	-Mean: 35 dys	-15 Medial -1 Lateral	-2 No -14 NR	-11 Female -5 Male	-Mean: 32	-2 yes -14 NR	-92 -87 -14 NR	-16 NR	-16 NR	-2 conservative -7 ORIF -2 ORIF > TKA -6 TKA	Case series, retrospective	Good
Campi et al. [16]	South Africa	522 cem 598 cem. less	NR	65	49	6	-NR	-6 Medial	-6 NR	-6 Female	-6 NR	-6 NR	-6 NR	-6 Oxford (Biomet)	-6 No	-4 ORIF -2 TKA	Case series, prospective	Good
Campi et al. [16]	UK / New Zealand	1000	NR	66	45	2	-1 mo -2 mo	-Medial -Medial	-No -No	-NR -NR	-NR -NR	-NR -NR	-NR -NR	-Oxford (Biomet) -Oxford (Biomet)	-No -No	-TKA -TKA	Case series, prospective	Good
Confalonieri et al. [22]	Italy	40	NR	70	53	1	-Intraop	-Medial	-No	-NR	-NR	-NR	-NR	-AMC (Corin)	-Yes	-ORIF	RCT, prospective	Fair
Costa et al. [23]	USA	34	30	73	44	4	-2 mo -3 mo -6 mo -18 mo	-Medial -Medial -Medial -Medial	-No -No -No -No	-Female -Female -Female -Male	-NR -NR -NR -NR	-Yes -Yes -Yes -No	-64 -78 -61 -NR	-EIUS (Stryker) -EIUS (Stryker) -EIUS (Stryker) -EIUS (Stryker)	-Yes -Yes -Yes -Yes	-TKA -TKA -TKA -TKA	RCT, prospective	Fair
Crawford et al. [24]	USA	576	32	62	59	10	-10 NR	- 10 Medial	-10 NR	-10 NR	-10 NR	-10 NR	-10 NR	-10 Oxford (Zimmer)	-10 NR	-10 TKA	Cohort study, retrospective	Fair
Darrith et al. [25]	USA	178	31	55	37	1	-NR	-NR	-NR	-Male	-NR	-NR	-68	-NR	-NR	-Conservative	Cohort study, retrospective	Fair
Epinette et al. [27]	France	NR	NR	NR	NR	15	-15 NR	-15 NR	-5 yes -10 No	-15 NR	-15 NR	-15 NR	-15 NR	-15 NR	-15 NR	-15 NR	Case series, retrospective	Fair
Forster et al. [28]	Australia	30	NR	67	53	1	-Intraop	-Lateral	-No	-Female	-NR	-Yes	-80	-Fixed-bearing	-Yes	-Conservative	Cohort study, prospective	Fair
Geller et al. [30]	USA	64	31	67	59	2	-NR -1 yr	-Medial -Medial	-No -Yes	-NR -NR	-NR -NR	-NR -NR	-NR -NR	-Fixed-bearings -Fixed-bearings	-Yes -Yes	-TKA -TKA	Cohort study, prospective	Good
Gesell et al. [31]	USA	47	NR	68	59	1	-10 dys	-Medial	-NR	-Medial	-NR	-NR	-NR	-Miller-Galante (Zimmer)	-Yes	-Conservative	Case series, retrospective	Good
Gill et al. [32]	UK	466	29	67	49	1	-3 mo	-Medial	-No	-Female	-NR	-NR	-77	-Physica ZUK (LIMA)	-Yes	-TKA	Case series, prospective	Good
Gleeson et al. [33]	UK	104	NR	66	50	1	-8 mo	-Medial	-No	-NR	-NR	-NR	-NR	-NR	-Yes	-TKA	RCT, prospective	Poor
Hamilton et al. [36]	USA	517	29	66	62	2	-5 mo -2 yrs	-Medial -Medial	-NR -NR	-NR -NR	-NR -NR	-NR -NR	-NR -NR	-Preservation (DePuy) -Preservation (DePuy)	-Yes -Yes	-TKA -TKA	Case series, retrospective	Good
Hamilton et al. [37]	USA	221	29	66	59	3	-2 mo -3 mo -14 mo	-Medial -Medial -Medial	-No -No -No	-Female -Male -Male	-33 -37 -27	-NR -NR -NR	-64 -61 -69	-Preservation (DePuy) -Preservation (DePuy) -Preservation (DePuy)	-NR -NR -NR	-TKA -TKA -TKA	Case series, retrospective	Good
Jeer et al. [43]	Australia	66	NR	69	NR	1	-2 wks	-Medial	-No	-Female	-NR	-NR	-64	-LCS (Depuy)	-No	-Consrevative > TKA	Case series, retrospective	Good
Ji et al. [44]	South Korea	246	NR	64	84	1	-Intraop	-Medial	-No	-NR	-NR	-NR	-NR	-Oxford (Biomet)	-Yes	-Consrevative > TKA	Case series, retrospective	Good
Kaneko et al. [45]	Japan	61	NR	74	73	4	-6 mo -7 mo -2 yr -5 yr	-Medial -Medial -Medial -Medial	-Yes -No -No -No	-Female -NR -NR -NR	-NR -NR -NR -NR	-NR -NR -NR -NR	-74 -NR -NR -NR	-Physica ZUK (LIMA) -Physica ZUK (LIMA) -Physica ZUK (LIMA) -Physica ZUK (LIMA)	-Yes -Yes -Yes -Yes	-TKA -Conservative -Conservative -Conservative	Case series, retrospective	Good
Kerens et al. [46]	Holland	60 cem. less 60 cem	30	63	51	2	-1 mo -2 mo	-Medial -Medial	-NR -NR	-NR -NR	-NR -NR	-NR -NR	-NR -NR	-Oxford (Biomet) -Oxford (Biomet)	-Yes -No	-TKA -NR	Cohort study, retrospective	Fair
Kim et al. [47]	South Korea	1576	NR	62	90	5	-Intraop -NR -NR -NR -NR	-Medial -Medial -Medial -Medial -Medial	-No -No -Yes -Yes -Yes	-Female -Female -Female -Female -Female	-NR -NR -NR -NR -NR	-NR -NR -NR -NR -NR	-NR -NR -NR -NR -NR	-NR -NR -NR -NR -NR	-NR -NR -NR -NR -NR	-ORIF -ORIF -ORIF -ORIF > TKA -TKA	Case series, retrospective	Good
Kim et al. [48]	South Korea	82	26	55	95	1	-7 yrs	-Medial	-NR	-Female	-NR	-NR	-60	-Oxford Phase 3 (Biomet)	-Yes	-TKA	Case series, retrospective	Good
Koh et al. [50]	South Korea	101	26	62	89	3	-NR -NR -NR	-Medial -Medial -Medial	-NR -NR -NR	-NR -NR -NR	-NR -NR -NR	-NR -NR -NR	-NR -NR -NR	-Fixed-bearing -Fixed-bearing -Fixed-bearing	-Yes	-NR	Cohort study, retrospective	Fair
Kort et al. [51]	Holland	154	NR	56	67	1	-4 wks	-Medial	-Yes	-NR	-NR	-NR	-NR	-Oxford Phase 3 (Biomet)	-Yes	-Conservative	Case series, prospective	Good
Lecuire et al. [53]	France	65	28	72	72	1	-Intraop	-Medial	-No	-NR	-NR	-NR	-NR	-Alpina (Biomet)	-No	-ORIF	Case series, retrospective	Good
Leenders et al. [54]	Holland	122	29	63	70	4	-1 mo -1.5 mo -4 mo -5 mo	-Medial -Medial -Medial -Medial	-NR -NR -NR -NR	-NR -NR -NR -NR	-NR -NR -NR -NR	-NR -NR -NR -NR	-NR -NR -NR -NR	-Oxford Phase3 (Biomet) -Oxford Phase3 (Biomet) -Oxford Phase3 (Biomet) -Oxford Phase3 (Biomet)	-No -No -No -No	-ORIF -Conservative -TKA -TKA	Case series, retrospective	Fair
Liddle et al. [55]	UK	1000	NR	66	43	4	-Intraop -Intraop -Intraop -Intraop	-Medial -Medial -Medial -Medial	-No -No -No -No	-Male -NR -NR -NR	-NR -NR -NR -NR	-NR -NR -NR -NR	-62 -NR -NR -NR	Oxford (Biomet) Oxford (Biomet) Oxford (Biomet) Oxford (Biomet)	-No -No -No -No	-Conservative -TKA -TKA -ORIF	Case series, prospective	Good
Lim et al. [56]	Singapore	263	26	63	72	1	18 mo	Medial	NR	-NR	NR	NR	NR	Fixed-bearing	-Yes	-TKA	Case series, prospective	Fair
Lindstrand et al. [57]	Sweden	123	NR	72	70	2	9 mo 13 mo	-Medial -Medial	-No -Yes	-Female -Female	-NR -NR	-NR -NR	-71 -77	-Fixed-bearing	-Yes -Yes	-NR -NR	Case series, prospective	Good
Lisowski et al. [58]	Holland	244	28	72	NR	1	-Intraop	-Medial	-No	-NR	-NR	-NR	-NR	-Oxford Phase 3 (Biomet)	-Yes	-Conservative	Case series, prospective	Good
Lombardi Jr et al. [59]	USA	115	31	61	63	2	-7 mo -22 mo	-Medial -Medial	-NR -NR	-NR -NR	-NR -NR	-NR -NR	-NR -NR	-Oxford Phase 3 (Biomet) -Oxford Phase 3 (Biomet)	-Yes -Yes	-TKA -TKA	Cohort study, retrospective	Good
Marya et al. [61]	India	29	NR	83	16	1	-Intraop	-Medial	-No	-Male	-NR	yes	87	-Allegretto (Zimmer)	-Yes	-ORIF	Case series, prospective	Good
Panzram et al. [70]	Germany	30	28	63	44	1	-1 mo	-Medial	-No	-NR	-NR	NR	NR	-Oxford (Biomet)	-No	-ORIF&UKA	Case series, retrospective	Good
Pongcharoen et al. [72]	Thailand	201	27	64	75	1	-3 mo	-Medial	-NR	-NR	-NR	NR	NR	-Oxford (Zimmer-Biomet)	-Yes	NR	Cohort study, retrospective	Good
Rajasekhar et al. [73]	UK	135	NR	70	57	1	-Intraop	-Medial	-No	-NR	-NR	NR	NR	-Oxford Phase 2 (Biomet)	-Yes	-ORIF	Case series, retrospective	Fair
Saxler et al. [77]	Germany	361	NR	70	67	1	-Intraop	-Medial	-No	-NR	-NR	NR	NR	-AMC (Corin)	-NR	-ORIF	Case series, prospective	Good
Schotanus et al. [54]	Holland	NR	NR	NR	NR	1	-7.1 yrs	-Medial	-NR	-Female	-NR	NR	58	-NR	-NR	-TKA	Case series, prospective	Good
Smith et al. 2012	UK	187	NR	65	68	1	-Intraop	-Medial	-NR	-NR	-NR	-NR	-NR	-Oxford Phase 3 (Biomet)	-Yes	-TKA	Case series, retrospective	Good
Song et al. [44]	South Korea	68	26	64	96	2	-5 wks -7 wks	-Medial -Medial	-No -No	-NR -Female	-NR -NR	-NR -Yes	-NR -76	-Miller-Galante (Zimmer) -Miller-Galante (Zimmer)	-Yes -Yes	-Conservative -TKA	Cohort study, prospective	Good
Song et al [85]	South Korea	100	26	66	87	2	-4 wks -NR	-Medial -Medial	-NR -NR	-NR -NR	-NR -NR	-NR -NR	-NR -NR	-Oxford Phase 3 (Biomet) -Oxford Phase 3 (Biomet)	-Yes -Yes	-TKA -TKA	Case series, retrospective	Fair
Thompson et al. [86]	USA	229	29	66	60	2	-18 dys -28 dys	-Medial -Lateral	-NR -NR	-Female -Female	-NR -NR	-NR -NR	-81 -68	-NR -NR	-Yes -Yes	-TKA -TKA	Case series, prospective	Fair
Vardi et al. [88]	UK	206	NR	64	37	5	-Intraop -6 wks -6 wks -6 wks -6 mo	-Lateral -NR -NR -NR -NR	-No -No -No -No -No	-NR -NR -NR -NR -NR	-NR -NR -NR -NR -NR	-NR -NR -NR -NR -NR	-NR -NR -NR -NR -NR	-NR -NR -NR -NR -NR	-NR -NR -NR -NR -NR	-ORIF > TKA -TKA -TKA -TKA -Conservative	Case series, retrospective	Fair
Weber et al. [89]	Germany	40	30	69	52	1	-6 wks	-Medial	-No	-Female	-NR	-Yes	-89	-Univation (Aesculap)	-Yes	-TKA	Cohort study, prospective	Good
Woo et al. [91]	Singapore	966	25	62	75	6	-1 mo -1 mo -1 mo -1 mo -1 mo -5 mo	-Medial -Medial -Medial -Medial -Medial -Medial	-No -No -No -No -No -Yes	-Female -Female -Female -Female -Female -Female	-19.3 -29.5 -24.3 -33 -22.5 -40.1	-Yes -Yes -No -No -No -No	-62 -58 -76 -67 -77 -65	-Fixed-bearing -Fixed-bearing -Fixed-bearing -Fixed-bearing -Fixed-bearing -Fixed-bearing	-Yes -Yes -Yes -Yes -Yes -Yes	-Conservative -Conservative -Conserative > ORIF -Conservative -Conservative -TKA	Case series, retrospective	Good
Yokoyama et al. [93]	Japan	167	NR	77	73	12	-12 NR	-12 Medial	-12 NR	-12 Female	-12 NR	-12 Yes	mean 79,4	-12 Fixed-bearings	-Yes	-11 Coservative -1 TKA	Case series, retrospective	Fair
Yoshida et al. [95]	Japan	1279	NR	77	82	3	-0.17 yrs -0.25 yrs -0.67 yrs	-Medial -Medial -Medial	-No -No -No	-NR -NR -NR	-NR -NR -NR	-Yes -Yes -No	-NR -NR -NR	-Oxford Phase 3 (Biomet) -Oxford Phase 3 (Biomet) -Oxford Phase 3 (Biomet)	-NR -NR -NR	-TKA -TKA -TKA	Case series, prospective	Good
Yoshikawa et al. [96]	Japan	156	NR	73	70	6	NR	-6 Medial	-6 NR	-6 Female	-6 NR	-6 NR	-6 NR	-Oxford (Biomet)	-6 No	-6 NR	Case series, retrospective	Fair

*UKA* unicompartmental knee arthroplasty; *BMI* body mass index; *ORIF* open reduction internal fixation; *NR* not reported; *TKA* total knee arthroplasty

*Consensus-based Clinical Case Reporting (CARE) checklist was used as a quality assessment tool

5.
